# Mentalising and depression: a mini-review on behavior, neural substrates, and treatment options

**DOI:** 10.3389/fpsyt.2023.1116306

**Published:** 2023-06-15

**Authors:** Benedikt P. Langenbach, Katja Koelkebeck, Daria Knoch

**Affiliations:** ^1^Department of Psychiatry and Psychotherapy, Faculty of Medicine, LVR University Hospital Essen, University of Duisburg-Essen, Essen, Germany; ^2^Center for Translational Neuro- and Behavioral Sciences, University of Duisburg-Essen, Essen, Germany; ^3^Department of Social Neuroscience and Social Psychology, Institute of Psychology, University of Bern, Bern, Switzerland

**Keywords:** depression, mentalising, social neuroscience, theory of mind, psychotherapy, TMS, pharmacotherapy

## Abstract

Major depression is one of the most common mental disorders, affecting millions of people around the globe. In recent years, researchers increasingly investigated social cognition in depression and discovered pronounced alterations. A special focus has been put on mentalising or Theory of Mind, the ability to recognize and understand another person’s thoughts and feelings. While there is behavioral evidence for deficits in this ability in patients with depression as well as specialized therapeutic interventions, the neuroscientific substrates are only beginning to be understood. In this mini-review, we take a social neuroscience perspective to analyse the importance of altered mentalising in depression and whether it can help to understand the origins and perpetuation of the disorder. We will put a special focus on treatment options and corresponding neural changes to identify relevant paths for future (neuroscientific) research.

## 1. Introduction

Over the course of their lifetime, 10–20% of the population will suffer from major depressive disorder (MDD) (1, 2). Risk of recurrence is high, with up to 35% of patients experiencing at least one further episode within 15 years (3). For up to 25% of patients, MDD takes a chronic course (4). While the core symptoms of MDD concern depressed mood, loss of energy, and negative thought patterns, researchers and practitioners increasingly turned to the concept of mentalising (i.e., the ability to infer thoughts and emotions of other people) to better understand some of the symptoms and to try to predict the course of the disorder. In this review, we will take a social neuroscience perspective to shed light on the role of (reduced) mentalising in depression. Section 2 gives a working definition of mentalising and briefly covers measurements and neural foundations of mentalising in healthy people. In Section 3, we discuss behavioral and neuroscientific findings regarding mentalising in depression, origins of impaired mentalising, and its predictive qualities. Finally, Section 4 covers different treatment options (psychotherapy, pharmacotherapy, and brain stimulation) and their behavioral and neural impact on mentalising. The discussion aims to identify relevant paths for future research.

## 2. Mentalising

Broadly speaking, mentalising, also called “perspective-taking,” can be defined as having a mental representation of another person’s mental states (e.g., thoughts and emotions). The word “mentalising” has been in use for well over a century (5), and is currently in use in areas as diverse as evolutionary biology, primate research, neuroscience, psychotherapy, or developmental psychology. Naturally, a concept that is prominent in such diverse areas will have multiple, sometimes diverging definitions. For example, some authors use mentalisation only to refer to the representation of affective states and “theory of mind” (ToM) to describe the representation of epistemic states (like beliefs or intentions) (6, 7). For others, ToM comprises both (8), sometimes with a differentiation between affective and cognitive ToM (9, 10). In this paper we follow the broader and common meaning, defining mentalising as the ability to recognize and form a mental representation of both affective and cognitive states of others while suppressing one’s own (11). Mentalising is crucial for basic processes like emotion recognition (12) as well as for successful social interactions and develops gradually during childhood (13). While a comprehensive review of mentalising in healthy people is beyond the scope of this article [but see (14)], we will here present key findings relevant for the understanding of (neural) alterations related to mentalising in MDD.

### 2.1. Measuring mentalising

There is a large number of tasks used to measure mentalising [for an overview, see (8)]. For example, basic visual perspective-tasking tasks ask participants to imagine taking the (physical) perspective of somebody else (15). Basic emotional tasks require participants to label emotions from pictures of faces or eyes (16). More complex tasks present participants with stories and ask for social comprehension [e.g., understanding a faux-pas (17)]. Finally, video-based tasks present social interactions and ask participants for thoughts, feelings, intentions or motives of the people in the film (18, 19). Recently, critique emerged about a number of mentalising tasks, questioning whether they actually require participants to form a mental model about somebody else’s mind (8). For example, one could argue that recognizing a facial emotion is possible without forming a mental representation of what another person is feeling or thinking (and why), and different social cognition tasks seem to represent slightly different aspects of underlying, hierarchical cognitive functions (20). Given these different approaches, it seems advisable to take the specific task into account when interpreting study results.

### 2.2. Mentalising and the “social brain”

As a complex social-cognitive function, mentalising is rooted in a large network of different brain areas. A recent meta-analysis found overlapping, but distinct neural networks for cognitive and affective mentalising (21): cognitive mentalising correlated with activation of the temporoparietal junction (TPJ), ventromedial prefrontal cortex (PFC), precuneus, inferior temporal gyrus and temporal pole, among others. Affective mentalising leads to activation in in the TPJ, middle temporal gyrus, precuneus and prefrontal areas, among others. Brain stimulation studies showed that disrupting the TPJ reduces people’s ability to mentalise with others or even with their own future self (22). Potentially, this is because the TPJ is required to differentiate one’s own thoughts and feelings from other people’s (23). Precuneus and medial PFC, on the other hand, might be important for attributing mental states to other people (21), and default mode network connectivity in the medial PFC correlates with social (dys)function (24). It has been suggested that affective mentalising recruits both empathy and cognitive mentalising (10), and this idea is indeed supported by the overlapping networks for these functions (21) and the fact that mentalising with another person increases empathic concern (25). Still, empathy and mentalising are discernible both neutrally and behaviorally.

### 2.3. Psychodynamic theories and reflective functioning

Importantly, the term mentalising (often used interchangeably with “reflective functioning”) is also used in the literature on psychodynamic psychotherapy, where it has a slightly broader meaning: here, it means the capacity to understand one’s own mental states as well as the mental states of others (26). Additionally, a high degree of mentalising requires people to have insight into *why* people behave the way they do (i.e., have insight into their own motives and potentially biographic explanations). Finally, a good mentaliser should acknowledge that mentalising can never be perfect. Reflective functioning is most often rated by a trained clinician, but a self-report questionnaire also exists (27). We here use mentalising in the narrower definition given in the previous section, but do discuss relevant psychodynamic research where it fits within the scope of this article (especially when discussing treatment options in Section 4.1).

## 3. Mentalising in depression

Traditionally, research on MDD has focused on the core emotional and cognitive symptoms such as low mood, loss of interest, loss of energy, reduced concentration, negative thought patterns or rumination. However, interpersonal relations and mentalising have also garnered substantial interest (28). Indeed, a recent meta-analysis established substantially reduced mentalising in MDD, for both cognitive and affective mentalising, and for both decoding tasks (e.g., emotion recognition) and reasoning tasks (e.g., identifying false-beliefs or intentions) (29, 30). For the affective component, these deficits might partly stem from a negative evaluation bias in MDD, so that patients with MDD are more likely to infer negative mental states (31, 32). Neither age nor gender seem to influence the degree of impairment, while people with more severe depressive symptomology show the largest deficits in mentalising (29, 30). Because studies showing the most pronounced deficits typically included patients with a chronic course of MDD, some authors have argued that mentalising deficits are particularly prominent in chronic depression (33). However, when directly comparing first-episode and chronic MDD, these differences apparently do not appear (34, 35). While chronicity therefore cannot be regarded as moderator of mentalising deficits, there is evidence that mentalising might be more strongly impaired in MDD with psychotic symptoms (36). Additionally, one might wonder whether specific subtypes of patients with MDD also show specific impairments (e.g., only relating to affective or cognitive mentalising). This sounds particularly prominent as MDD is somewhat of an umbrella category, and the pathogenesis and biographical details differ vastly. However, concrete evidence for specific subtypes regarding mentalising is sparse and overall, both affective and cognitive mentalising seemed to be impaired in MDD. As of yet, the only reliable finding regarding subtypes seems to be that mentalising deficits correlate with severity of symptoms, with more severely depressed patients showing the most pronounced deficits in mentalising, and sub-clinical samples showing no mentalising deficits at all (29). These findings, however, extend to mentalising overall, and there is no evidence for a specific pattern of mentalising deficits.

One might also speculate about the influence of comorbid disorders on mentalising: for example, disorders like social phobia or eating disorders seem to correspond to over-mentalising (37, 38), yet at the same time often occur together with MDD. As, however, over- and under-mentalising can co-occur in the same individual (39), these findings might be less puzzling than one might think (38). Still, it remains entirely possible that different subgroups exhibit specific patterns (e.g., patients who suffered from depressive symptoms since adolescence vs. patients who developed them only later in life). Until more research is conducted, however, any findings on depression subtypes should be interpreted as preliminary results, and we do agree with Bora’s and Berk’s opinion (30) that more research into mentalising in different subtypes of MDD is needed.

Given the high number of patients with MDD with comorbid personality disorders, one might wonder whether it is the personality disorders, not MDD, that actually leads to decreased mentalising. This might sound even more plausible because by its very definition, personality disorders are characterized by impaired self-functioning or interpersonal functioning (40). However, a number of studies showed that deficits in mentalising also occur in MDD when controlling for personality disorders (41, 42) or when excluding patients with personality disorder altogether (43, 44). Thus, is seems valid to assume that reduced mentalising in MDD is not just confounded by comorbid personality disorders.

As most studies on mentalising in MDD are cross-sectional, they cannot inform us on whether reduced mentalising is a result of MDD, or whether it precedes the disorder and thus constitutes a risk factor or even cause of MDD. There have been, however, some theoretical attempts to explain a potential causal effect of mentalising on MDD. For example, in the context of CBASP, it is assumed that (chronically) depressed patients exhibit (child-like) preoperational thinking patterns, struggle with “true empathy,” and are “pervasively egocentric.” This is supposed to lead to frustrating interpersonal situations that do not satisfy one’s interpersonal needs, which ultimately would lead to the development and maintenance of depressive symptoms (45). Another explanation is the idea that reduced theory of mind leads to a pattern of social interaction that might drive other people away, with the resulting social isolation promoting depressive symptoms (46). Additionally, there is evidence that deficits in ToM persist after remission: Inoue et al. (47) showed that patients with remitted MDD performed worse than matched controls in a false-belief task (but no differences were observed in factual observations during the task). These results are supported by Ladegaard et al. (48), who showed impaired social cognition (including ToM) but normal cognitive functioning. Additionally, stronger deficits in ToM predict both a higher risk of relapse and more severe symptoms in MDD (49, 50). Thus, it seems likely that reduced mentalising contributes to MDD, and is not merely a consequence. It should be noted, however, that the opposite route of action has also been discussed. For example, it has been proposed that a negativity bias and constant rumination in MDD lead to impaired mentalising because less cognitive resources are available to accurately interpret social cues, or that social withdrawal typical to MDD leads to less “practice” of mentalising and social skills (29, 51).

There are only few neuroscientific studies investigating mentalising in patients with MDD. Lai et al. (52) showed altered functional connectivity in a network comprising areas that have been related to ToM before, such as the left precentral gyrus, left angular gyrus, bilateral rolandic operculum and left inferior frontal gyrus. However, as these brain areas are involved in many cognitive processes, it remains speculative whether alterations are actually related to altered ToM. Converging evidence stems from studies on postpartum depression: using near-infrared spectroscopy, Morgan et al. could analyse brain connectivity during a naturalistic mother–child interaction (reading a book together) (53). Mothers with more severe depressive symptoms showed a lower connectivity between the right TPJ and lateral PFC, but greater connectivity between the right TPJ and the anterior medial PFC. The authors interpret their results as indication that during mentalising about their infant’s thoughts and feelings, mothers with MDD might be less able to express and regulate their own emotions, but better at engaging in emotional bonding with their infants, although this remains somewhat speculative.

At least partly, deficits in mentalising and corresponding neural alterations seem to be rooted in negative experience early in life. Indeed, childhood maltreatment is not only a risk factor for (chronic) depression (54), it also predicts lower mentalising later in life (55, 56). Additionally, Hentze et al. (55) showed that childhood maltreatment correlates positively with amygdala activation during an affective ToM task. This is in line with research showing that childhood maltreatment leads to heightened amygdala responsiveness as well as reduced gray matter in insula, orbitofrontal cortex, anterior cingulate gyrus, superior temporal sulcus, middle temporal lobe, and caudate (57).

The fact that childhood maltreatment predicts both mentalising and MDD gives further support to the idea that mentalising might precede MDD and that reduced mentalising might pose a vulnerability for MDD, potentially mediated through neural changes in the amygdala system. While the amygdala might not be a prototypical part of the mentalising system, it has been argued that the amygdala is involved in fast, automatic mentalising (58) and patients with amygdala damage show impairments in mentalising (59). This might be particularly true if the mentalising task at hand involves emotion recognition and processing, whereby the mentalising system might require output from the amygdala (60).

Interestingly, neural alterations are also visible in individuals with high familiar risk for MDD. Using a family network analysis, Abraham et al. (61) showed that individuals with heightened familiar risk exhibited reduced influence of the inferior frontal gyrus and posterior superior temporal gyrus within a social cognition network. Additionally, these neural alterations predicted both depressive symptoms and deficits in interpersonal adjustment at an 8-year follow-up.

Taken together, there seems to be clear evidence that mentalising is reduced in MDD and that patients with reduced mentalising are at higher risk of recurrence, that mentalising deficits correspond to pronounced changes in the “social brain” and that these cognitive and neural alterations can at least partly be matched unto early-life stressors.

## 4. Treatment options targeting mentalising

### 4.1. Psychotherapy

There is a plethora of psychotherapeutic approaches that address peoples’ cognition and emotions toward others. Most prominent probably, there are psychodynamic therapy (with a focus on unresolved inner conflicts), cognitive-behavioral therapy (with a focus on the learning history and resulting dysfunctional thought patterns) and interpersonal psychotherapy (with a focus on the ability to assert needs and wishes in interpersonal relationships). Differences in treatment success between all types of psychotherapy are small for MDD (62), although psychodynamic therapy might potentially be least effective (63). Regardless of therapy type, though, less than 40% of patients with MDD respond to psychotherapy (62). Most psychotherapeutic interventions do not have an explicit focus on mentalising, but focus on depressive symptoms at large. Still, mentalising is being addressed by a number of them, sometimes implicitly. Among all types of therapy, interpersonal psychotherapy most directly addresses interpersonal relations and tries to aid patients with managing social situations and it has been proposed that mentalising constitutes a potential mechanism of change in interpersonal therapy (64). Indeed, a randomized trial with 96 patients showed that even though both cognitive-behavioral therapy and interpersonal therapy were effective for the treatment of MDD, only the latter led to an improvement in mentalising (65). Standard cognitive-behavioral therapy (CBT) puts a strong focus on automatic and maladaptive thought patterns, challenging patients’ implicit and explicit assumptions. Because these automatic thoughts can also relate to other people (e.g., “my colleagues have not contacted me since I’m in the hospital, that’s because they do not like me”), it has been proposed that CBT targets mentalising almost by default (66, 67), even though enhancing mentalising is normally no explicit goal of CBT. However, empirical support for the assumed improvements in mentalising is lacking. Similarly, proponents of psychodynamic treatments (which aim to give patients insight into inner conflicts affecting their lives) have argued that mentalising is particularly prone to change through psychodynamic therapies (33). Empirical evidence, however, is somewhat mixed, with some studies showing improvements of mentalising (68), others showing no effect (69) or an effect only at a later follow-up (70). Additionally, neither of the studies were placebo-controlled. This might pose a problem especially for the studies showing an improvement: not only is it unclear what caused the changes, but by the nature of the research design, the researchers who rated reflective functioning could not have been blinded [for an overview of other psychotherapy studies using reflective functioning but not as outcome variable (e.g., as predictor of therapy success), see also (33)]. In addition to these “classic” approaches, there are also a number of specialized therapeutic interventions with a stronger focus on mentalising. One CBT approach is the “cognitive behavioral analysis system of psychotherapy” [CBASP, (45)]. It assumes that chronically depressed patients often lack the belief that their behavior will elicit an (emotional or behavioral) response in others and therefore remain inactive. Thus, therapy sessions focus on the analysis of what the patient did (or did not) do to achieve their interpersonal goals, with the explicit goal of fostering mentalising. A recent review concludes that CBASP might be more effective than both treatment-as-usual and interpersonal psychotherapy (71). However, we are not aware of a study that directly measures the effect of CBASP on an established mentalising task—which is somewhat surprising since the method claims to improve depressive symptoms via improvement of mentalising skills. The most noteworthy psychodynamic approach with an explicit focus on mentalising is mentalisation-based therapy [MBT, (72)], aiming to improve both the understanding of others as well as one self. MBT has been shown to be effective for a range of disorders, although it might be less effective for the treatment of MDD than third-wave cognitive-behavioral therapy (73). Still, more research is needed to make conclusive statements. Finally, there are trainings that do not aim to address depressive symptoms at large, but specifically target mentalising, but they are still somewhat niche (74). Unfortunately, neuroscientific studies of changes in mentalising elicited by psychotherapy are relatively rare, but some first evidence suggests neural changes in the mentalising system following psychotherapy. For example, in a small study on 10 patients, a 12-week CBASP-therapy enhanced amygdala reactivity toward emotional faces (75), which might indicate that neural emotion processing is altered after therapy. Additionally, there is some evidence from studying patients with bipolar disorder in remission: Meyer et al. (76) investigated the impact of a program with 24 h of CBT, targeting impulse regulation, ToM, and social skills. Not only did the intervention stabilize patients, they also showed increased activity in what the authors called the “ToM network” (bilateral TPJ, posterior cingulate cortex, precuneus) during a ToM task. However, the neural changes were not accompanied by improved ToM performances in the task and ToM performance was unrelated to clinical outcomes. Thus, the clinical relevance of the neural changes remains somewhat unclear. Additionally, we do not know whether the results generalize to patients with MDD, even though this seems likely. Comparatively more research has dealt with the question of neural changes following psychotherapy without a specific focus on mentalising. Indeed, meta-analytic evidence shows decreased activation in the insula and the anterior cingulate cortex following psychotherapy for MDD and/or anxiety (77). Another meta-analysis found that psychotherapy (compared to pharmacotherapy) led to more activity in the medial PFC (78), which is prominently involved in the mentalising system. Thus, one might speculate whether these changes affect mentalising, but more evidence is needed.

### 4.2. Pharmacotherapeutic effects on mentalising

There have been theories about a potential mode of action of (serotonergic) antidepressants through social cognition, mainly based on the involvement of serotonergic systems in social cognition (79). However, concrete evidence for a positive effect of pharmacotherapy on mentalising is scarce: there are studies showing an improvement in emotion recognition after starting antidepressant medication (80, 81), but they are not placebo-controlled. Thus, it remains unclear whether improvements actually stem from the medication or from other factors such as the placebo effect or regression to the mean (82). Additionally, changes in mentalising capacity were visible long before the antidepressant effects, which might indicate that changes were due to re-test effects rather than actual changes caused by the drugs, although this remains speculative. On a neural level, pharmacotherapy seems to lead to alterations in amygdala activity (78), which is involved in emotion processing (but many other processes as well, see above). Apart from traditional antidepressants, there has been some interest into the effects of oxytocin as a potential treatment in MDD. It has long been speculated whether the oxytocin system is altered in MDD (83), but a recent meta-analysis showed now difference in endogenous oxytocin levels between patients with MDD and healthy controls (84) and evidence for an antidepressive effect of (exogenous) oxytocin is lacking (85). Still, oxytocin seems to improve mentalising in other disorders [(86, 87), but see also (88)] and oxytocin has pronounced effects on neural areas involved in social cognition, including the medial PFC, insula, and caudate (89). Thus, it might influence mentalising in MDD, but further research is warranted. Other drugs have also been discussed for the treatment of MDD, including ketamine, psychedelics, and amphetamines. However, because their use is still rare or even experimental, and because evidence of effects on ToM in MDD is lacking or shown to be absent (90), further research is needed before any conclusions can be drawn. In sum, it is currently somewhat unclear whether (antidepressant) drugs alter the mentalising system of patients with MDD.

### 4.3. Brain stimulation

In recent years, transcranial magnetic stimulation (TMS) has become an effective and increasingly popular treatment for MDD (91). Typically, patients receive a series of TMS sessions, during which their cortex (typically the dorsolateral PFC; dlPFC) is stimulated using electric current produced by a coil placed on their scalp. There is good evidence that TMS can reduce mentalising (9, 92, 93), yet studies showing improved mentalising following TMS are rare [but do exist, see (90, 94)]. To our knowledge, there are only two studies testing the influence of TMS on mentalising in MDD, and none using neuroscientific techniques. A pilot study without control-group on 14 patients (95) showed no overall effect of a 4-week TMS treatment at the dlPFC on mentalising (specifically, emotion recognition). There was, however, a statistically significant interaction between the improvement on core depressive symptoms and mentalising. Similarly, a larger, sham-controlled study on 120 patients (96) showed improvements in both cognitive and affective mentalising after a 4-week TMS treatment. Specifically, patients showed improvements in emotion recognition as well as improvements in inferring the intentions behind indirect speech utterances or hints. Thus, TMS treatment does indeed seem to improve mentalising in MDD. However, it should be noted that treatment also improved general (non-social) cognitive abilities. Thus, it is possible that TMS did not specifically affect mentalising but that patients’ improved cognitive functions influenced the mentalising task (e.g., because they were able to concentrate better or were more alert), especially since it is known that overall cognitive functioning affects mentalising both in healthy people (97) as well as other mental disorders (98). It therefore remains unclear whether TMS can be used to specifically enhance mentalising in MDD, although it does seem to have a positive effect overall. Another type of brain stimulation, transcranial direct current stimulations (tDCS), works by applying a low electrical current directly to a person’s scalp, with the aim to increase or decrease the functioning of underlying brain areas. While not routinely used in the treatment of MDD, there is some indication that tDCS to the dlPFC might alleviate depressive symptoms (99, 100). Additionally, there is evidence that tDCS to the TPJ (101, 102), but also the dlPFC (103) enhances mentalising. Thus, it might well be that tDCS could be used to enhance mentalising in MDD, but further research is needed to provide empirical support for this assumption. For both TMS and tDCS, protocols for MDD typically focus on the dlPFC, yet there are other areas with more prominent connections to mentalising, such as medial PF or TJP. It might therefore be worth investigating whether different stimulation locations could have positive effects on mentalising in MDD.

## 5. Discussion

Mentalising capacity is substantially impaired for many patients with MDD. These deficits seem to stem at least partly from negative early-life experiences (e.g., childhood maltreatment) and correspond to neural alterations to the mentalising network (e.g., TPJ, inferior frontal gyrus) and connected structures (e.g., amygdala). MDD is currently treated with a number of different approaches, yet evidence of effects on mentalising are rare. However, a number of psychotherapeutic approaches have started to focus implicitly or explicitly on mentalising to improve patients’ well-being. In light of the evidence today, we believe that mentalising might be a prominent target to improve the course of MDD and prevent recurrence of the disorder. However, several aspects remain unclear and should be investigated in future research. In particular, it might be worth identifying whether a causal link between reduced mentalising (before onset of the disorder) and MDD does exist, although this would require large longitudinal data sets. Whether strengthening mentalising can reduce risk of relapse or even improve well-being of patients in acute phases would be another valuable line of research. Comparisons of different types of mentalising, and comparisons of different groups of patients (e.g., first episode MDD vs. chronic depression) are still rare and might be worthwhile investigating. Similarly, one might wonder whether patients that developed depressive symptoms as reactions to acute life-stressors but did not suffer from negative events during childhood are similarly affected in terms of mentalising capacity. It might also be helpful to conduct more longitudinal research, to better understand when reduced mentalising (or social cognition more broadly) is a cause for MDD, and when it can also be a consequence of it. Finally, much of the existing knowledge stems from research from countries of the Global North, even though culture influences the role of social cognition in mental disorders (104). Getting a better understanding of the role of reduced mentalising for different patient populations might help to tailor more targeted interventions. Because mentalising deficits sometimes persist after the end of an acute depressive episode (47), but also predict future recurrence of MDD, investigating mentalising training as relapse prevention after successful therapy would be very informative. Because much is still unknown about the role of mentalising in MDD, researchers might want to include at least a basic mentalising measure in future intervention studies. From a neuroscientific point of view, it could be interesting to not only measure neural alterations in patients with MDD, but also changes following targeted interventions. Ultimately, of course, patients with MDD seek help not because they want to improve their mentalising abilities, but because they suffer from the core depressive symptoms. Thus, improving mentalising will likely not be a target in and of itself, but rather a tool to achieve better quality of life. While the existing evidence warrants caution, taking a stronger focus on (improving) the mentalising capacity of depressed patients might still be a promising lever for change.

## Author contributions

BPL and DK developed the concept of the manuscript. BPL wrote the first draft. DK and KK read and complemented the manuscript. All authors contributed to the article and approved the submitted version.

## Funding

This work has partially been funded by the Typhaine Foundation.

## Conflict of interest

The authors declare that the research was conducted in the absence of any commercial or financial relationships that could be construed as a potential conflict of interest.

## Publisher’s note

All claims expressed in this article are solely those of the authors and do not necessarily represent those of their affiliated organizations, or those of the publisher, the editors and the reviewers. Any product that may be evaluated in this article, or claim that may be made by its manufacturer, is not guaranteed or endorsed by the publisher.
